# Bridging the Gap in Graduate Medical Education: A Longitudinal Analysis of Medical and Fellowship Director Experiences in Telehealth

**DOI:** 10.7759/cureus.31947

**Published:** 2022-11-27

**Authors:** Ragan A DuBose-Morris, Chris Pelic, Ryann Shealy

**Affiliations:** 1 Education, Medical University of South Carolina, Charleston, USA; 2 Psychiatry, Medical University of South Carolina, Charleston, USA; 3 Medicine, Medical University of South Carolina, Charleston, USA

**Keywords:** program director, clinical training, telehealth education, graduate medical education, curriculum

## Abstract

Background

With the evolving nature of telehealth services being supported across graduate medical programs, understanding changing attitudes among program directors is essential for training future physicians.

Objective

This 5-year longitudinal survey analysis provides details regarding the knowledge, skills, competency, and confidence levels present among program directors and the ways telehealth training (didactic and experiential) supports resident education.

Methods

A longitudinal observation survey was sent to 77 program directors (48% completion) from an academic health system. Data were compared from survey responses from 2016 to 2021 with additional information collected about current training processes in 2021. Paired sample t-tests, quantitative data analysis, and qualitative thematic analysis results are reported based on a convenience sampling of all program and fellowship directors.

Results

Results show that while telehealth knowledge and use increased among program director faculty, systemic concerns about the feasibility of telehealth from a time and cost perspective remain high. In 2016 and 2021, most of those surveyed (28.9% and 37.1%, respectively) were concerned about reimbursement. Directors remain committed to telehealth resident education, with over 60% stating that it is "essential to future practice". The qualitative thematic analysis highlighted the need for additional resources and support to conduct telehealth and the variability within disciplines of the use, therefore modeling, of telehealth in clinical settings.

Conclusions

The overall utilization of telehealth by graduate medical education programs has increased along with the continued need for training to prepare residents for current and future practice.

## Introduction

Telehealth is an essential aspect of the future of healthcare. It has been crucial to many hospitals and practices during the COVID-19 pandemic - especially in its use to support Graduate Medical Education (GME) programs [1]. Defined as the remote provision of healthcare through telecommunication tools [2], telehealth encompasses a range of methods for connecting providers and patients through real-time audio and video, text-based messaging, and remote monitoring applications. Although its use has increased over the years, telehealth adoption has yet to reach its full potential. Known barriers to using telehealth include lack of training/knowledge and lack of reimbursement, especially among primary care providers [3-4].

Since the inception of formal GME training for telehealth, overall utilization of telehealth has increased along with the continued need for training to prepare trainees for current and future practice [5-6]. Multiple professional and accrediting bodies, including the Association of American Colleges (AAMC) [7] and the American Board of Pediatrics (ABP) [8], have recently released Entrustable Professional Activities specific to telehealth services. The Medical University of South Carolina is one of the few institutions in the country that had formal GME training for telehealth before COVID-19, over a significant time and with multiple programs participating - although the need is now being recognized by accreditors [9]. Formal training programs at MUSC have historically spanned three academic years and included didactic, experiential, and clinical service experiences [5]. The curriculum has been compressed to prepare residents and fellows for telehealth consultations as early as Program Year 1. By surveying internal GME programs, we were able to determine the current state of training and propose adjustments to offerings to meet the rapidly evolving needs of medical directors and trainees.

These findings were previously presented at The Society for Education and the Advancement of Research in Connected Health (SEARCH) 2021 National Telehealth Research Symposium.

## Materials and methods

To meet the educational and clinical goals for GME programs, we conducted a longitudinal survey of current residency and fellowship program directors on their self-assessed knowledge and confidence in practicing and supervising telehealth encounters. College of Medicine residency and fellowship program directors were surveyed in 2016 using the RedCap platform [10-11]. An observational follow-up survey was sent to all 77 directors across 25 programs, with 37 directors completing the survey (48%) in 2021 (please see Figures 3-5 in the Appendices). The 2016 and 2021 surveys were identical except for additional trainee-specific questions posed in 2021 to determine the amount of training received by the learners and their integration into telehealth services under the supervision of GME faculty. Questions were developed originally in 2016 based on validated undergraduate medical and interprofessional student surveys administered each semester to gauge baseline knowledge, skills and confidence. Clinical education faculty reviewed and approved the survey as part of a sub-group tasked by leadership. All residency and fellowship program directors were invited to respond to the surveys through email invitations sent to the official GME listserv for the academic health system. Two reminder invitations were sent to directors following the initial request over one month.

SPSS [12] standard procedures were applied to analyze the survey data. Data were compared from 2016 to 2021 with additional information about current resident/fellow training processes being analyzed for the 2021 data set. Paired sample t-tests were used to compare mean scores by survey year. Five-point Likert scores (i.e., not at all/could write a book) were analyzed for all survey question/statement data points. Inductive qualitative data analysis was conducted on educational modality, duration, and topic area related to trainee initiatives. The study team analyzed responses from optional open-ended survey questions related to telehealth adoption and trainee training processes for relevant themes. Thematic familiarization, coding, definition, and write-up were conducted utilizing a narrative qualitative research design. The institutional review board determined that this project was exempt.

## Results

Program and fellowship director respondents represent providers in various practice locations across the academic institution and are primarily based in hospitals or on-campus clinics, as detailed in Table 1.

**Table 1:** Program and Fellowship Director Practice Demographics

	Count, %	Count, %
My primary practice setting is the:	2016	2021
Hospital	6, 35.3%	17, 45.9%
Clinic (on campus)	10, 58.8%	11, 29.7%
Clinic (off campus)	0, 0.0%	6, 16.2%
Emergency Department	0, 0.0%	1, 2.7%
Virtual Only (2021 Survey Only)	N/A	1, 2.7%
Other	1, 5.9%	1, 2.7%

**Figure 1:**
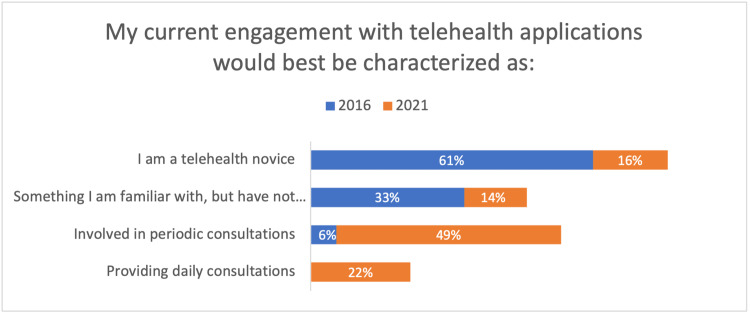
Directors' Telehealth Engagement

As expected, between 2016 and 2021, directors gained knowledge regarding telehealth implementation and their actual telehealth practice levels (Figure 1).

There is a significant difference between 2016 and 2021 in the residency directors' comfort in demonstrating/explaining telehealth tools (p=0.043) (Table 2).

**Table 2:** I feel comfortable demonstrating/explaining at least three telehealth tools (p=0.043)

	2016 (n=17)	2021 (n=37)
No way	9, 52.9%	8, 21.6%
Maybe	3, 17.6%	11, 29.7%
I could demonstrate/explain 1 tool	3, 17.6%	5, 13.5%
I could demonstrate/explain 2 tools	1, 5.9%	9, 24.3%
I could demonstrate/explain 3 tools	1, 5.9%	4, 10.8%

**Figure 2:**
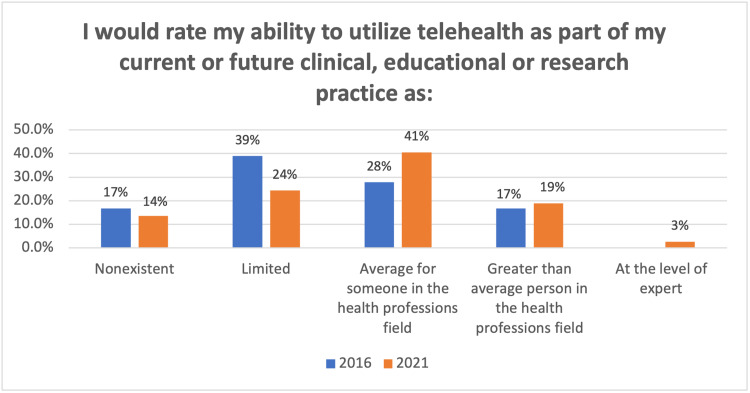
Directors' ability to utilize telehealth as part of future practice (p=0.039):

**Figure 3:**
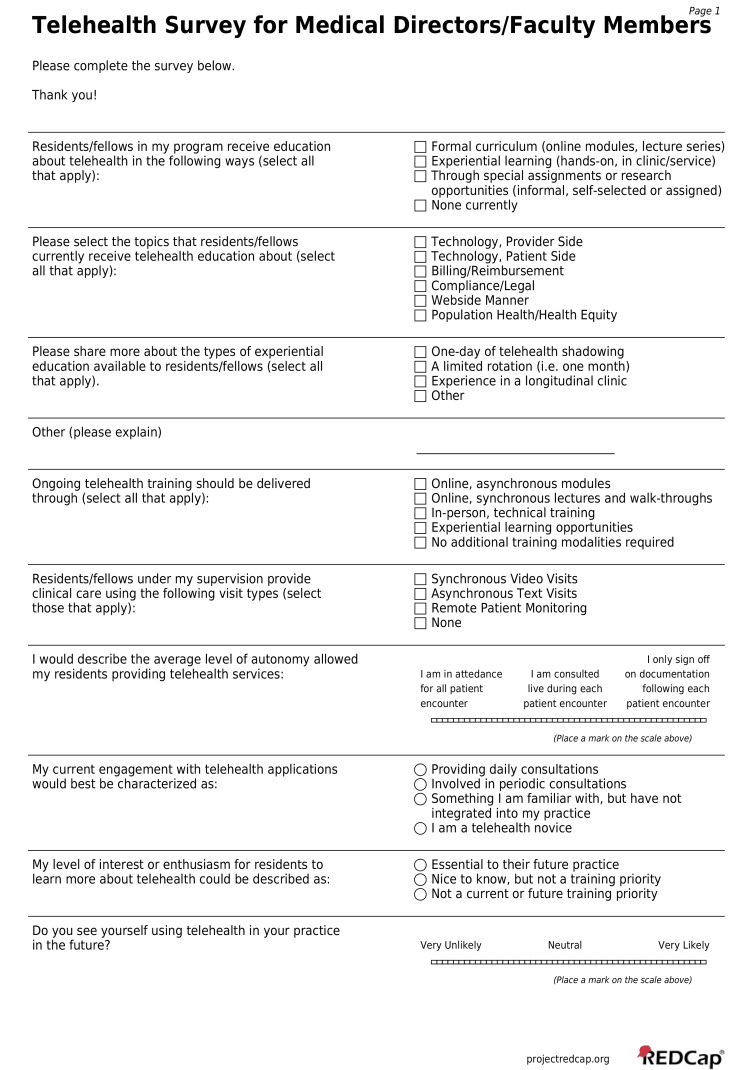
Supplement - 2021 Telehealth Survey for Medical Directors/Faculty Members: Page 1

**Figure 4:**
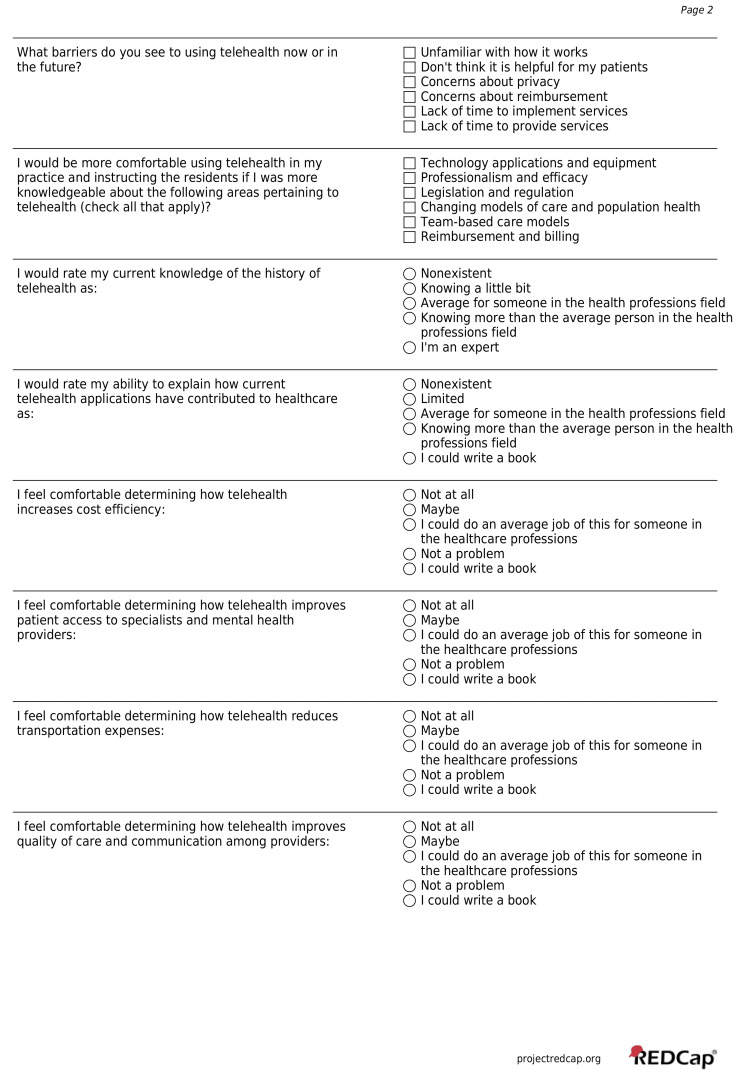
Supplement - 2021 Telehealth Survey for Medical Directors/Faculty Members: Page 2

**Figure 5:**
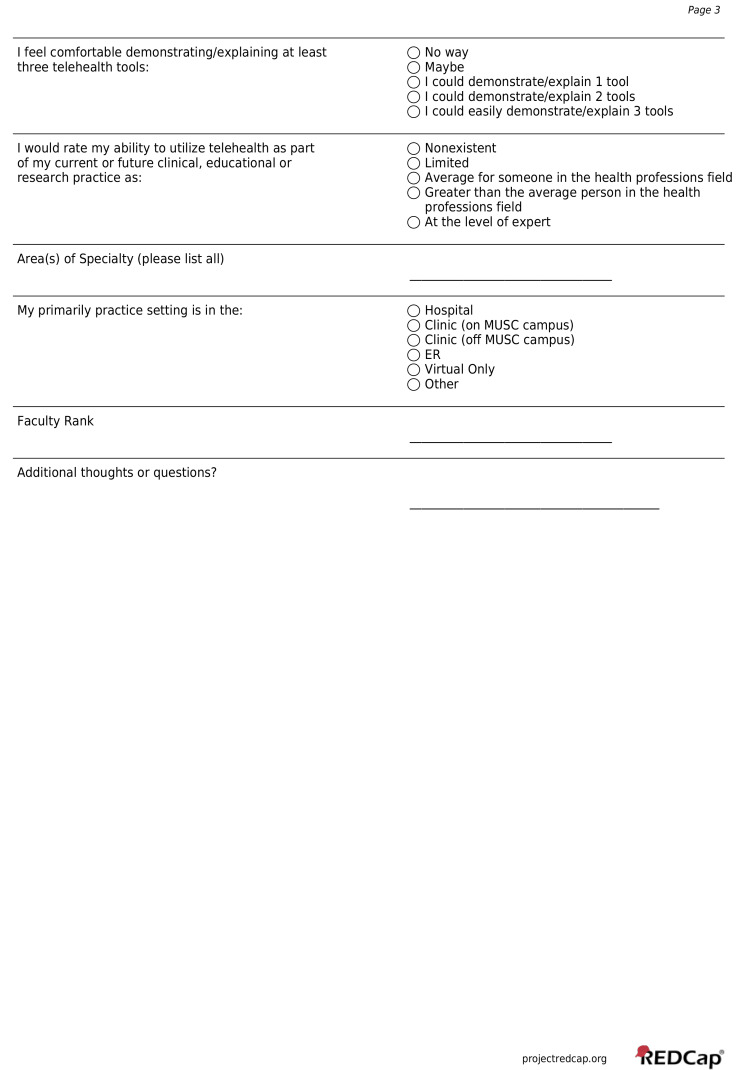
Supplement - 2021 Telehealth Survey for Medical Directors/Faculty Members: Page 3

In addition, significant changes were reported in directors' ability to utilize telehealth in current or future clinical, educational, or research practice (p=0.039) (Figure 2). This data visualization shows increased familiarity and confidence based on real-world utilization.

Telehealth knowledge and use are both subject to systemic barriers. In 2016 and 2021, most programs surveyed (28.9% and 37.1%, respectively) were concerned about reimbursement as a significant barrier to telehealth, as shown in this descriptive comparison of total responses (Table 3). Also consistently of concern is the lack of time to provide services via telehealth (24.4% and 19.4%).

**Table 3:** Program and Fellowship Director Barriers and Additional Telehealth Knowledge Needs

		2016 Count (n=17), %	2021 Count (n=37), %
What barriers do you see to using telehealth now or in the future? (All that apply)						
Concerns about reimbursement		13, 28.9%	23, 37.1%
Lack of time to provide services		11, 24.4%	12, 19.4%
Lack of time to implement services		9, 20.0%	9, 14.5%
Unfamiliar with how it works		7, 15.6%	9, 14.5%
Concerns about privacy		4, 8.9%	4, 6.5%
Don't think it is helpful for my patients		1, 2.2%	6, 9.7%

Additional information from the 2021 survey shows that programs are modeling the use of telehealth in clinical settings, with 75.6% of trainees represented in this survey providing care under supervision through synchronous video visits (63.4%), remote patient monitoring activities (9.8%), and asynchronous text visits (2.4%). Overall, trainees primarily receive instruction through experience in a longitudinal clinic (73.3%) and through one-day (6.7%) or limited rotations (6.7%) that might last up to a month.

Directors' enthusiasm for residents and fellows to learn more about telehealth remained consistent, with 61.1% in 2016 and 62.2% in 2021 stating it is "essential to their future practice". Respondents report that trainees were more likely to be trained through experiential clinic offerings (70.3%) than in online or in-person didactics (29.7%). Still, 24.3%, or nine programs, reported no training being provided in 2021. For programs providing education (n=26), multiple topics are being covered, including provider-side technologies (80.8%); billing/reimbursement (57.5%); compliance/legal (42.3%), and webside manner (30.8%). Directors feel that ongoing training modalities should be provided in the following ways: online, asynchronous modules (23.2%); online, synchronous lectures and walk-throughs (20.3%); in-person, technical training (30.4%) and experiential learning opportunities (34.8%).

Finally, open-ended responses provided additional context to the types of education and clinical services being provided by the programs. A thematic analysis of the open-ended prompts noted two main themes: the practice and instruction related to telehealth are highly variable by discipline, and the general need for more resources and support to provide telehealth limits what can be modeled. Some nuances in the training continuum were noted, including this one statement: "As program director, I am responsible for the curriculum of telehealth, but a different provider does the supervision exclusively, and I do not have first-hand experience with that care delivery." These types of structural challenges within training programs warrant additional consideration.

## Discussion

Some programs ran into limitations with telehealth when implemented to scale during COVID-19, and questions still need to be answered about the long-term capacity for telehealth consultations [4]. With further telehealth training to increase physician knowledge and comfort, these barriers could be decreased and allow patients to better access telehealth as part of a menu of services. Although increases in overall telehealth knowledge are likely due to the surge of telehealth consultations provided during the COVID-19 pandemic by faculty and trainees alike [13], only one respondent to the 2021 survey considered themselves an expert on their ability to utilize telehealth as a part of their current or future clinical, educational, or research practice. The lack of respondents who believe they are an expert in telehealth shows room to improve telehealth education while aligning future training with competencies [14].

The consistent percentage of respondents who consider telehealth essential to future practice (61.1% vs. 62.2%) likely reflects concerns about reimbursement and the time constraints needed to implement services in the current environment. While not statistically significant, program directors' comfort in determining how telehealth increases cost efficiency shows an average to less than average comfort level, with only 17% "knowing more than the average person in the health professions field" (p=0.068). Currently, the percentage of directors considering telehealth essential continues to be constrained due to regulatory and reimbursement advances. This is an area for additional provider engagement, stakeholder education, and health policy intervention to achieve a consistent policy roadmap that informs faculty and trainee development.

The survey helped identify potential areas of curricula reform for GME programs that aligns with a shift to competency-based education in telehealth [14]. Incorporating telehealth into formal GME programs could help bridge this knowledge gap and lead to more prepared physicians, as seen in national needs assessments for telehealth education across learner levels [13-15]. Overall improvements in health outcomes are also expected, including access to, quality of, and equity of care for patients through telehealth services [16]. Few of the surveyed programs and fellowship directors did not use telehealth in their clinical practice due to the nature of their specialties. However, telehealth is increasingly seen as an appropriate technology to support at least minimal healthcare functions across multiple practice settings [17].

Limitations of the study included the discrepancy between the number of programs responding to the survey over time and the lack of longitudinal data by specialty. Additionally, the opinions were that of the program directors and not necessarily of the faculty training the residents and fellows. The initial survey responses in 2016 were provided by a small subset of specialties inclined to consider incorporating telehealth into their training programs and clinical operations. The respondents and their affiliated programs increased participation in the 2021 offering, most likely due to increased use and awareness of telehealth in the intervening years. The research question did not attempt to draw direct comparisons between specific programs longitudinally due to the low initial response rate in 2016. Program directors were selected as respondents as decision-makers informed about curriculum integration, but future studies could include the full faculty team by specialty.

Further investigations of program director knowledge, comfort, and practice levels in relationship to the training provided for trainees are warranted within specific specialties and institutions as part of the internal continuous quality improvement process. Additionally, this study was limited in examining one academic health system's GME leadership. Although the institution has had a broad implementation of telehealth over many years, results from this research might not be generalizable to other GME programs or academic health systems. Internally, this data has been used to refine curriculum offerings across program years based on program support.

Given the compression of didactic and experiential training due to the pandemic, additional opportunities for curriculum integration are needed to support in-person education and provide workforce support for GME programs investing in telehealth [6,15]. Regardless of discipline, additional training and support are needed for GME faculty to improve their knowledge and confidence with the provision of training on telehealth processes [13]. These ongoing curriculum improvements will serve the evolving need of future providers and their patients.

## Conclusions

Since the inception of formal GME training for telehealth in 2016 at MUSC, the overall utilization of telehealth has increased along with the continued need for training to prepare trainees for current and future practice. This study indicates that continuous telehealth policy adjustments are needed in areas of billing and reimbursement, and clinical workflow support is needed for programs to integrate telehealth fully. Similar surveys at the GME level can help identify curricular needs or reform. These structural changes and continued curriculum integration for trainees will ensure that the gap between current and future telehealth practice can be better addressed through formal didactic and experiential education methods.
